# Characterization of a Glycolipid Synthase Producing α-Galactosylceramide in *Bacteroides fragilis*

**DOI:** 10.3390/ijms232213975

**Published:** 2022-11-12

**Authors:** Marc Caballé, Magda Faijes, Antoni Planas

**Affiliations:** Laboratory of Biochemistry, Institut Químic de Sarrià, University Ramon Llull, 08017 Barcelona, Spain

**Keywords:** glycosyltransferase, glycosphingolipids, galactosylceramide

## Abstract

Glycolipids are complex molecules involved in important cellular processes. Among them, the glycosphingolipid α-galactosylceramide has proven to be of interest in biomedicine for its immunostimulatory capabilities. Given its structural requirements, the use of ceramide glycosyltransferase enzymes capable of synthesizing this molecule under in vivo or in vitro conditions is a potential production strategy. Several GT4 enzymes from *Bacteroides fragilis* were considered as potential candidates in addition to the known BF9343_3149, but only this one showed glycolipid synthase activity. The enzyme was expressed as a SUMO fusion protein to produce soluble protein. It is a non-processive glycosyltransferase that prefers UDP-Gal over UDP-Glc as a donor substrate, and maximum activity was found at pH 7.3 and around 30–35 °C. It does not require metal cations for activity as other GT4 enzymes, but Zn^2+^ inactivates the enzyme. The reaction occurs when the ceramide lipid acceptor is solubilized with BSA (100% conversion) but not when it is presented in mixed micelles, and anionic lipids do not increase activity, as in other membrane-associated glycolipid synthases. Further protein engineering to increase stability and activity can make feasible the enzymatic synthesis of α-GalCer for biomedical applications.

## 1. Introduction

Glycosphingolipids (GSLs) are glycoconjugates that are widely distributed among eukaryotic cells but less common in bacteria. These amphiphilic molecules consist of an acylated sphingoid base (ceramide, Cer) linked to a hydrophilic carbohydrate moiety. They are structurally diverse with regard to the acyl chain (length and modifications) and the sugar moiety attached to the ceramide, from monosaccharides to complex glycans (such as in gangliosides) [1]. GSLs are components of eukaryotic plasma membranes that are not uniformly distributed but clustered in “lipid rafts” (small lateral microdomains of self-associating membrane molecules) and play important roles in modulating receptor proteins in signal transduction pathways [1,2]. Bacterial GSLs are less common, mainly found in α-proteobacteria. Whereas eukaryotic GSL biosynthesis pathways are well characterized [1], bacterial GSL biosynthesis is less studied. A serine-palmitoyl transferase (SPT), which catalyzes the first step of ceramide synthesis, was identified from *Sphingomonas paucimobilis* [3], and some glycosyltransferases that transfer sugar to ceramide acceptor have been reported in some species, such as Sgt1 and Sgt2 in *Caulobacter crescentus* [4]. Some GSL-containing bacteria lack lipopolysaccharides, suggesting that bacterial GSL may be a functional replacement in their outer membrane [5].

In eukaryotic GSLs, the first sugar attached to the ceramide is typically a β-linked galactose (βGalCer) or glucose (βGlcCer). Several glycosyltransferases (GT) responsible for the first transfer to ceramide have been characterized (i.e., the ceramide glucosyltransferase UGCG [6]) as transferring the UDP-sugar donor to the ceramide acceptor with inversion of configuration. A relevant function of GSL in the human immune system is the activation of invariant natural killer T (iNKT) cells. iNKT cells are involved in the suppression of autoimmune reactions, cancer metastasis, and the graft-rejection response. Some GSLs presented by CD1d (an MHC class I molecule) of dendritic cells can activate a T-cell antigen receptor expressed on the cell surface of iNKT cells, in order to trigger the secretion of several cytokines (such as interleukin IL-2, IL-4 and interferon-γ) as important effectors in the subsequent immune response [7].

iNKT cells can also be activated by non-mammalian αGalCer, with an α-glycosidic linkage not found in mammals. Indeed, agelasphin, first isolated in 1993 as an anti-tumor agent in the marine sponge *Agelas mauritianus*, was identified as αGalCer [8,9]. Chemical optimization of the ceramide portion afforded a αGalCer congener with C18-phytosphingosine and cerotic acid (26:0), named KRN700, as a candidate for clinical applications [10]. More recently, αGalCer was identified as a lipid antigen for iNKT cells when presented by CD1d [11,12]. In the search of other natural sources, αGalCer was isolated from *Bacteroides fragilis*, a common component of the human colon microbiota; it is generally commensal but can become pathogenic, causing infections if displaced into the bloodstream or surrounding tissues following trauma, surgery or disease [13]. αGalCer and other bacterial GSLs are exogenous ligands of CD1d and play important roles in the host’s immune system. The bacterial origin of αGalCer supports the hypothesis that the identified congeners in the sponge *Agelas* were actually produced by symbiotic bacteria [7]. 

Recently, Okino et al. [14] identified a glycosyltransferase from *Bacteroides fragilis* that can catalyze the reaction of αGalCer synthesis. This protein showed homology to ceramide UDP-glucuronosyltransferase (Cer-GlcAT) from *Z. mobilis*, another transferase that uses ceramide as an acceptor. They studied different substrates for the *B. fragilis* GT, showing that ceramide, but apparently not diacylglycerol, was the acceptor, and UDP-galactose or UDP-glucose (with preference for the first) were the donor substrates, but not UDP-GalNAc, UDP-GlcA, UDP-GlcNAc or GDP-Man. The α configuration of the glycosidic linkage confirmed the enzyme as a retaining glycosyltranferase synthesizing αGalCer. Other congeners have also been identified in other gut bacteria, such as *Bacteroides vulgatus* and *Prevotella capri* [15]. The *Bacteroides* αGalCer is thought to be a critical signaling molecule in gut physiology. Studies with mice identified a different congener of αGalCer with a β-hydroxylated palmitic acid and C18-sphinganine (αGalCerMLI) in the mice colon that was not detected in germ-free mice [16]. Since its production was dependent on diet and inflammation, it was suggested that its effector function through iNKT cells is important in gut energy and immunity. Although the synthetic pathway for this unique αGalCer is unknown, the identified *Bacteroides* GT may be involved in its production.

α-GalCer has gained attention as a vaccine adjuvant for the immunotherapy of tumors, by the induction of potent natural killer cell-dependent anti-tumor cytotoxic responses [17,18,19,20]. Since the chemical synthesis of α-GalCer is tedious with low yields [21,22], biotechnological production by biocatalysis and cell factory approaches are of current interest, thus, demanding better knowledge of its biosynthetic enzymes. To obtain further insights into the biosynthesis of αGalCer in *B. fragilis*, here, we search for other potential GTs with GSL synthase activity in the *B. fragilis* genome, and biochemically characterize the GT recently identified by Okino et al. [14]. 

## 2. Results

### 2.1. Bioinformatics Search of GT Candidates in the B. fragilis Genome

**Figure 1:**
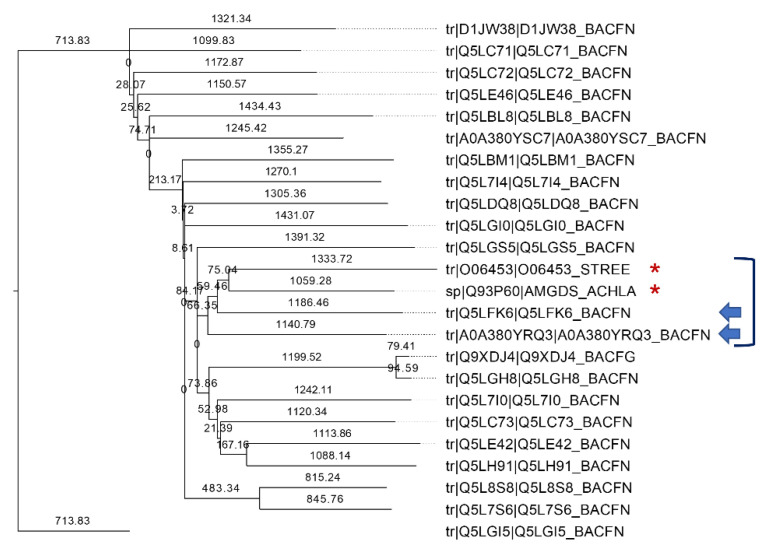
Neighbor-joining phylogenetic tree of annotated GT4 enzymes from *Bacteroides fragilis* and query sequences of monoglucosyldiacylglycerol synthases from *Acholeplasma laidlawii* (Uniprot Q93P60) and *Streptococcus pneumoniae* (UniProt O06453) (marked with *). The closest *B. fragilis* sequences are indicated with arrows.

Gene BF9343_3149 from *B. fragilis* was recently identified as encoding a glycolipid synthase (αCerGal_GT) by Ito and collaborators (Okino et al. [14]). The protein sequence shares 23.8% identity with *Z. mobilis* glucuronyl glycosyltransferase, and it was proven to catalyze the reaction between UPD-Gal and ceramide with a retaining mechanism. To obtain insight into glycolipid synthases in *B. fragilis*, we searched for GTs from this microorganism that could also show galactosylceramide transferase activity using the CAZy database, which compiles identified and annotated GTs [23]. A total of 83 putative GT genes from *B. fragilis* NCTC9343 are annotated in this database; however, only retaining GTs (families 3, 4, 5, 8 and 35) were considered in the analysis. GT3, 5 and 35 members were discarded, since these families contain enzymes involved in glycogen and starch metabolism. Families 4 and 8 contain enzymes that use lipids as acceptors, but only GT4 enzymes were considered since it is the only family with reported GT activities using ceramide or diacylglycerol as acceptors. The 22 annotated GT4 sequences from the *B. fragilis* genome were aligned with two biochemically characterized GT4 glycolipid synthases: the non-processive monoglucosyldiacylglycerol synthases from *Acholeplasma laidlawii* [24,25] and *Streptococcus pneumoniae* [25]. The results from the multiple sequence alignment (Supplementary Figure S1) are shown in the form of a neighbor-joining tree in Figure 1. Among the *B. fragilis* GT4 sequences, only two were clustered with the query sequences: the already identified BF9343_3149 (UniProt A0A380YRQ3), and BF9343_1306 (UniProt Q5LFK6). Next, the other four GT genes from *B. fragilis* among the non-classified GT sequences in CAZY were also considered: BF9343_3589 (UniProt Q5L962), BF9343_0008 (UniProt Q5LJ89), BF9343_0585 (UniProt Q5LHL6) and BF9343_0009 (UniProt A0A380YSP3). BLAST searches with these six candidate sequences were performed against the Swiss-Prot, RefSeq and TrEMBL databases. Candidates with high homology (≥50%) to other enzymes with known activity were discarded. For example, BF9343_0008 and BF9343_0009 showed high homology with D-inositol 3-phosphate glycosyltransferase (57.5% and 74.2%, respectively), and thus, were rejected. By contrast, BF9343_3589 and BF9343_0585 did not show any significant hits, and BF9343_1306 and BF9343_3149 showed homology (40% and 70%, respectively) to uncharacterized enzymes involved in cell wall synthesis or capsular polysaccharide biosynthesis. Therefore, the GT4 sequence BF9343_1306 and non-classified GT sequences BF9343_3589 and BF9343_0585 were considered as potential candidates with ceramide glycosyltransferase activity, in addition to the known BF9343_3149 (αCerGal_GT).

### 2.2. Identification of α-Galactosylceramide Transferase Activity in B. fragilis GT Candidates

**Figure 2:**
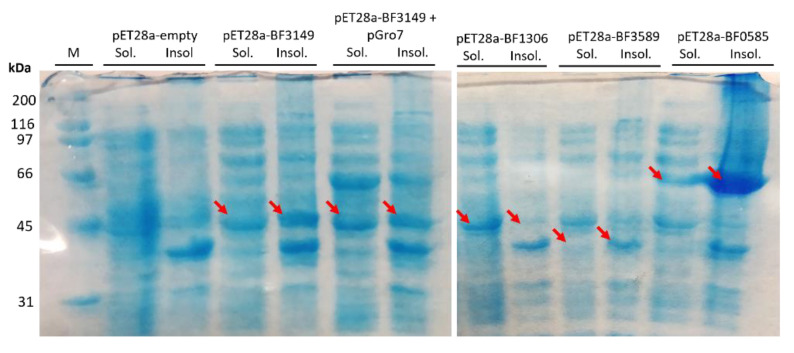
SDS-PAGE gel electrophoresis results of expression of *B. fragilis* GT candidates in *E. coli*. Labels indicate the soluble and insoluble fraction for each culture; M: protein molecular mass marker. Arrows show the expressed proteins. Expected MWs for BF3149, BF1306, BF3589 and BF0585 proteins were 45.0, 45.5, 40.4 and 63.8 kDa, respectively.

**Figure 3:**
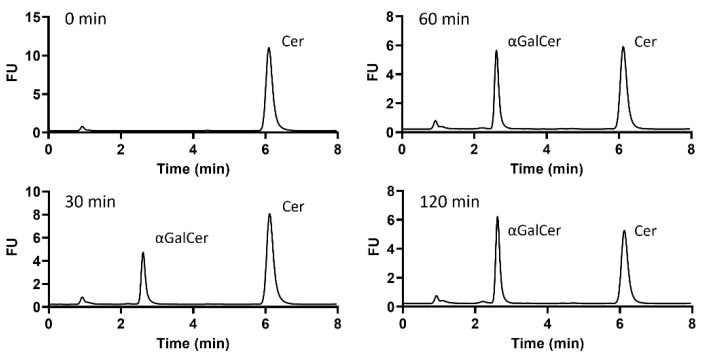
Time-course reaction monitoring of galactosyltransferase activity of a cell extract expressing BF9343_3149. The reaction was carried out with 25 µM of Cer-NBD in the presence of equimolar BSA, 1.25 mM of UDP-Gal and, as enzyme source, a cell-free extract from *E. coli*/pET28a-BF3149 (≈4 mg/mL total protein) in phosphate buffer at pH 7.5 and 37 °C.

The three GT candidates and BF9343_3149 were cloned into pET28a fused with C-terminal His-tag and expressed in *E. coli* cells for activity identification. Cultures were induced with IPTG at 25 °C and cell pellets were resuspended in phosphate buffer and lysed. The cell extracts and pellets from BF9343_3149 and BF9343_0585 presented the expected new proteins with molecular masses of 45.0 and 63.8 kDa, respectively (Figure 2). By contrast, the expression of BF9343_3589 and BF9343_1306 proteins with theoretical masses of 40.4 and 45.5 kDa were not clear compared to the control (empty pET28a). Nevertheless, all four cell extracts were assayed with UDP-Gal and Cer-NBD (C6-ceramide with fluorescent label, see Materials and Methods) for galactosyltransferase activity on ceramide. In this assay, the Cer-NBD acceptor is solubilized with an equimolar amount of BSA, the cell extract is added, and reactions are initiated by the addition of UDP-Gal at pH 7.5, 37 °C, and monitored by HPLC. Only the cell extract expressing BF9343_3149 showed activity synthesizing the corresponding galactosylceramide (Figure 3).

The results confirm that only BF9343_3149, but not BF9343_1306, BF9343_3589 and BF9343_0585, is a glycosylceramide synthase. 

### 2.3. Expression and Purification of B. fragilis Glycosyltransferase

The BF9343_3149 amino acid sequence was analyzed to identify possible transmembrane domains or signal peptide sequences to be removed for recombinant protein expression. TMHMM 2.0 [26,27] showed no transmembrane domains in the sequence, and SignalP 5.0 [28] did not predict any signal peptides either. The full-length protein was expressed as a C-terminal His-tagged protein in a pET28a vector. Expression in *E. coli* was explored at different conditions, since yield with standard IPTG induction at 25 °C was low, and the protein mainly remained in the insoluble fraction after cell lysis. Induction at different temperatures and co-expressions with pGro7 vector [29] encoding for GroEL and GroES chaperones was assayed, but the expression yields did not improve. Therefore, preparative protein expression was performed with IPTG induction at 25 °C with no chaperone co-expression. 

Solubilization of the cell extract to isolate solubilized protein for purification was further explored. The cells were lysed by sonication in different buffers. Phosphate buffer alone and containing CHAPS, Tris buffer with DTT, and HEPES buffer containing CHAPS and glycerol were assayed. Only cell extract with buffers without detergent presented activity, indicating that the detergent could interfere with the active enzyme. 

**Figure 4:**
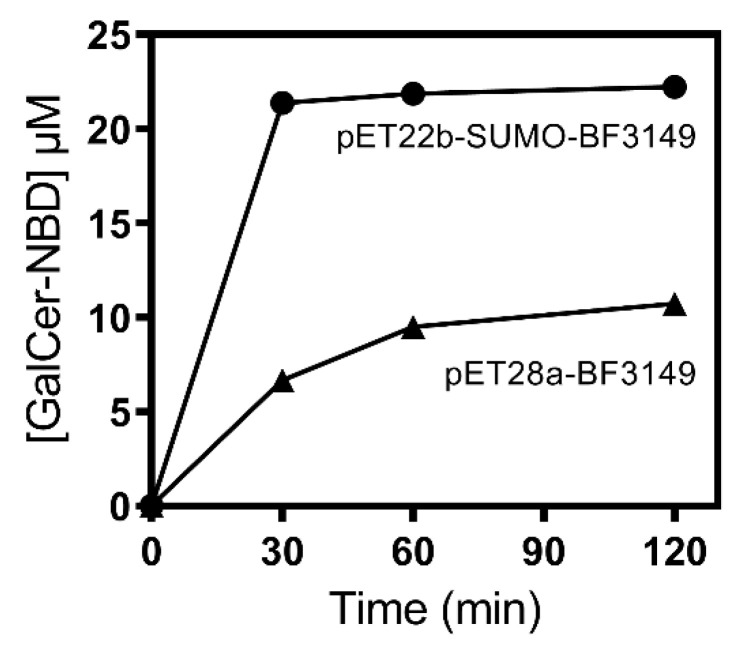
Ceramide-NBD activity assay of αGalCer_GT (BF9343_3149) cell-free extracts from different expression systems. Reactions were carried out with 25 µM of Cer-NBD in the presence of equimolar BSA, 1.25 mM of UDP-Gal and cell-free extracts of pET28a-BF9343_3149 and pET22b-SUMO-BF9343_3149 cultures (total protein concentration in the assay 9.9 ± 0.4 mg/mL), in 50 mM of phosphate buffer and 150 mM of NaCl at pH 7.5 and 37 °C.

Despite the presence of active enzyme in the soluble cell extract with phosphate buffer, the purification by metal affinity chromatography did not work. Since activity was only found in the flowthrough and not in the elution step with imidazole, it was thought that the His-tag was not exposed or was hydrolyzed. Then, the BF9343_3149 gene was subcloned into a pET22b expression vector fused with a different affinity tag, Strep-tag at the C-terminus, as well as the SUMO protein at the N-terminus trying to enhance protein solubility, as reported for other proteins [30,31]. Using this new vector, expression was performed with 0.1 mM of ITPG induction at 30 °C for 4 h. The cell-free extract in phosphate buffer showed 3.5 times higher activity than the previous one (Figure 4), proving the effectiveness of the SUMO fusion protein. In addition, purification using Strep-trap affinity chromatography was successful. The eluted protein after dialysis was about 0.39 mg/mL, with an overall yield of 0.6 mg per liter of culture.

### 2.4. Kinetic Characterization 

#### 2.4.1. Specific Activity

**Figure 5:**
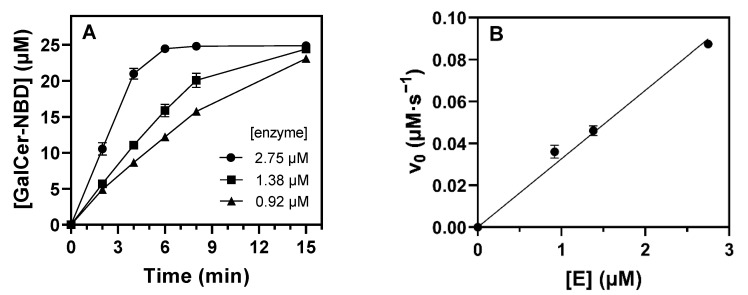
(**A**) Time course of αGalCer_GT reaction. (**B**) Linear dependence of initial rates with enzyme concentration. Reactions were carried out with 25 µM of Cer-NBD in the presence of equimolar BSA, 1.25 mM of UDP-Gal and 1 to 3 μM enzyme in 50 mM of phosphate buffer and 150 mM of NaCl at pH 7.5 and 37 °C.

Enzyme activity was determined at 1.25 mM of UDP-Gal donor and 25 µM of Cer-NBD acceptor solubilized as a complex with BSA in equimolar concentration (Cer-NBD:BSA 1:1) in phosphate buffer, pH 7.5, 37 °C. Time-course monitoring by HPLC showed that the enzyme forms αGalCer as a unique product and the reaction is complete after 15 min (Figure 5A and Figure S2). The specific activity was calculated to be 3.27·10^−2^ s^−1^ from the linear dependence of initial velocity with enzyme concentration (Figure 5B).

**Figure 6:**
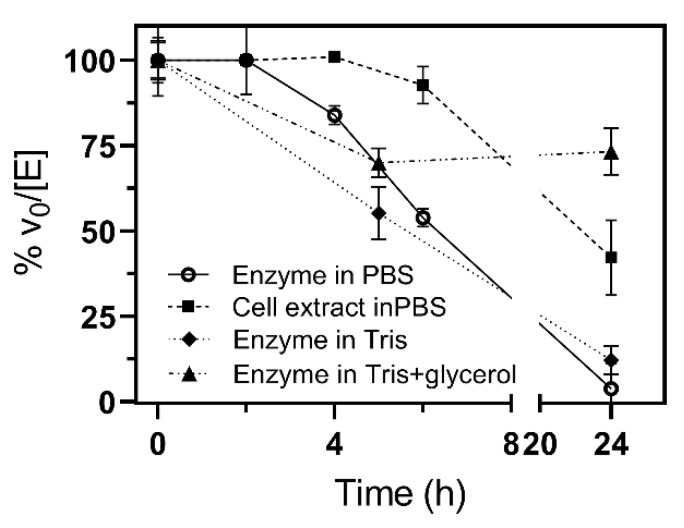
Stability of αGalCer_GT. The specific activity of freshly prepared enzyme stored at 4 °C in phosphate, pH 7.5, was measured at different times. Assay conditions: 25 µM of Cer-NBD in the presence of equimolar BSA, 1.25 mM of UDP-Gal and 1.39 µM of purified enzyme or soluble cell extracts (0.77 µM of active enzyme), in 50 mM of phosphate or Tris buffer and 150 mM of NaCl at pH 7.5 and 37 °C.

Unexpectedly, the stability of the purified enzyme was a key issue. Enzymatic activity was only completely maintained for 2 h, about 39% at 6 h and nearly lost at 24 h. This behavior was similar in Tris buffer and in the presence of 20% glycerol, while in the cell extract, the enzyme was stable until 5 h and kept 42% of activity after 24 h (Figure 6). Therefore, all activity data presented are for freshly expressed and purified protein (less than 2 h storage after purification).

#### 2.4.2. Metal Binding

**Figure 7:**
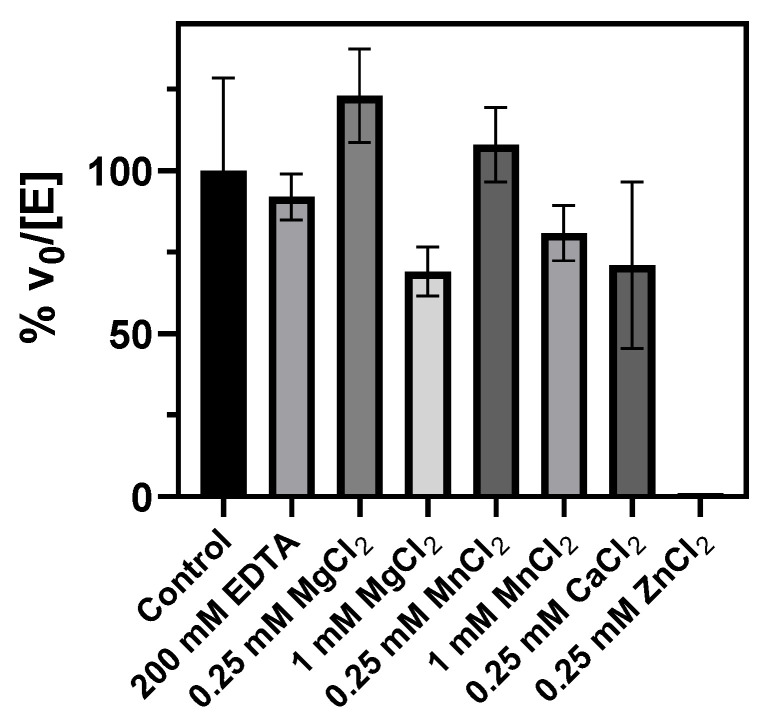
Effect of metal cations on αGalCer_GT activity. Specific activities (v_0_/[E]) were compared and plotted as % activity relative to the activity of freshly purified enzyme (control). Reaction conditions: 25 µM of Cer-NBD in the presence of equimolar BSA, 1.25 mM of UDP-Gal and 35–70 nM of enzyme in 20 mM of Tris buffer and 200 mM of NaCl at pH 7.5 and 37 °C.

Since cation requirement is common in glycosyltransferases, metal binding was also studied. Buffers used for protein purification and activity assays did not contain added metal cations. Treatment with EDTA did not reduce the activity relative to untreated and freshly purified enzyme, indicating that the enzyme either does not bind divalent cations or that the metal cation is strongly bound to the active site. Mg^2+^ and Mn^2+^ at 0.25 mM slightly increased the enzyme activity, but the effect was reduced at a higher concentration, with some inhibition at 1 mM of Mg^2+^, which reduced the activity to 70%. Whereas Ca^2+^ at 0.25 mM did not significantly affect the enzyme activity, Zn^2+^ inactivated the enzyme (Figure 7).

#### 2.4.3. Optimal pH and Temperature

**Figure 8:**
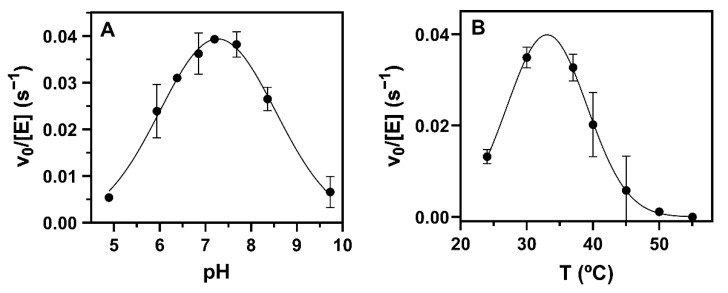
Optimal pH and temperature profiles of αGalCer_GT activity. Reactions were carried out with 25 µM of Cer-NBD in the presence of equimolar BSA, 1.25 mM of UDP-Gal and different enzyme concentrations. (**A**) pH profile between 5 and 9.5 with 50 mM/50mM of citrate/phosphate buffers adjusted at each pH, 37 °C and 830 nM of enzyme. (**B**) Temperature profile between 25 and 55 °C in phosphate buffer at 7.5 and 1.1–3.2 µM of enzyme.

The pH profile of specific activity using phosphate–citrate buffers follows a bell-shaped curve with the optimum catalytic efficiency at a pH of around 7.3 (Figure 8A). Between 6 and 8.5, the enzyme retained more than 70% activity, while it was essentially inactive at pH < 5 and >9.5. In addition, αCerGal_GT remained catalytically active from 25 °C to 40 °C with an optimal temperature around 30 °C, and it was inactivated at 50 °C (Figure 8B).

#### 2.4.4. Kinetic Parameters for Donor and Acceptor Substrates

**Figure 9:**
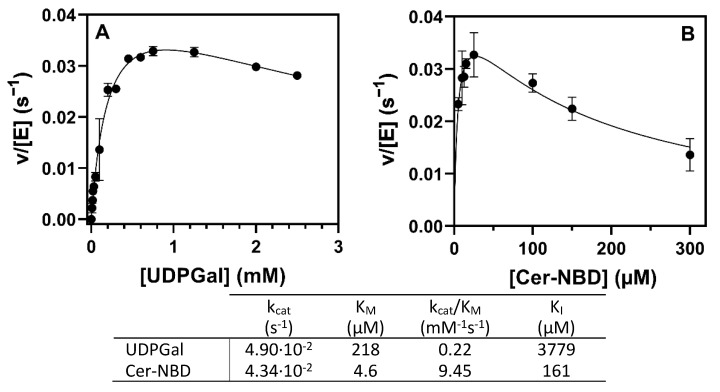
Kinetics of αGalCer_GT. (**A**) Michaelis–Menten curve for UDP-Gal. Reactions conditions: 10–2500 µM of UDP-Gal, 25 µM of Cer-NBD in the presence of equimolar BSA, 0.2–0.5 μM of enzyme, at pH 7.5 and 37 °C. (**B**) Michaelis–Menten curve for Cer-NBD. Reactions conditions: 1.25 mM of UDP-Gal, 5–300 µM of Cer-NBD in the presence of equimolar BSA, 0.35–0.75 μM of enzyme, at pH 7.5 and 37 °C.

Kinetics varying UDP-Gal at a saturating Cer-NBD concentration (25 µM) solubilized with BSA, and varying Cer-NBD concentration at a saturating UDP-Gal concentration (1.25 mM) at pH 7.5 and 37 °C, obeyed Michaelis–Menten kinetics with some substrate inhibition (Figure 9). K_M_ for UDP-Gal is 218 µM, with a catalytic efficiency of 0.22 mM^−1^·s^−1^, and a slight donor substrate inhibition can be observed above 2 mM. For the NBD-ceramide acceptor at saturating donor conditions (1.25 mM), kinetics show substrate inhibition with maximum activity at 25 µM of Cer-NBD, K_M_ of 4.6 µM and K_I_ of 161 µM.

In addition, UDP-Gal presents 14.5-fold higher specific activity than UDP-Glc, using NBD-ceramide as an acceptor at a 1.25 mM/25 µM donor/acceptor ratio (Figure S3). Assuming saturating conditions for both donors and acceptor, UDP-Gal is a better donor than UDP-Glc in terms of k_cat_.

#### 2.4.5. Activity in Mixed Micelles and in the Presence of Anionic Lipids

**Figure 10:**
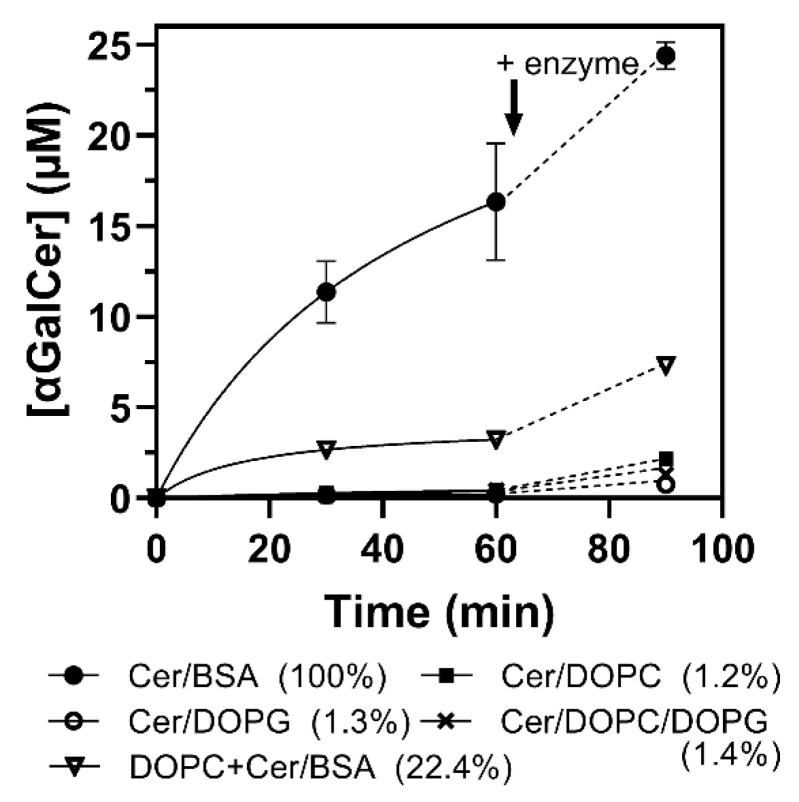
Reaction monitoring of αCerGal_GT activity using ceramide in mixed micelles as acceptor. Reactions were carried out with 25 µM of Cer-NBD, 1.25 mM of UDP-Gal, 0.25 µM of enzyme. Ceramide was solubilized with BSA or in mixed micelles with DOPC (1 mM) or DOPC/DOPG 0.6/0.4 (total 1 mM) or DOPG (1 mM). Moreover, activity was measured with Cer-NBD in BSA in the presence of DOPC (1 mM). Legend (in parenthesis): % activity at 30 min. At 60 min reaction, a fresh aliquot of enzyme was added (indicated with an arrow).

The αCerGal_GT kinetic characterization described above was performed with Cer-NBD solubilized with BSA. Since other bacterial and plant glycolipid synthases present activity on lipid micelles and require anionic phospholipids for activation [24,32,33], the effect of presenting the acceptor in micelles was evaluated using Cer-NBD with DOPC and/or DOPG mixed vesicles (DOPC, dioleoylphosphatidylcholine as neutral lipid, and DOPG, dioleoylphosphatidylglycerol as anionic lipid). After the incubation of Cer-NBD in micelles with the enzyme for 30 min, UDP-Gal was added. As can be observed in Figure 10, enzyme activity decreases considerably in ceramide/DOPC or DOPG micelles compared with CerNBD solubilized with BSA. Neither the presence of neutral or anionic lipids in vesicles activates the enzyme, but rather inhibits it, since the addition of Cer-NBD solubilized with BSA to the enzyme preincubated with DOPC vesicles only results in 22% of the original activity.

### 2.5. Modeled αGalCer_GT Structure and Ligand Binding

**Figure 11:**
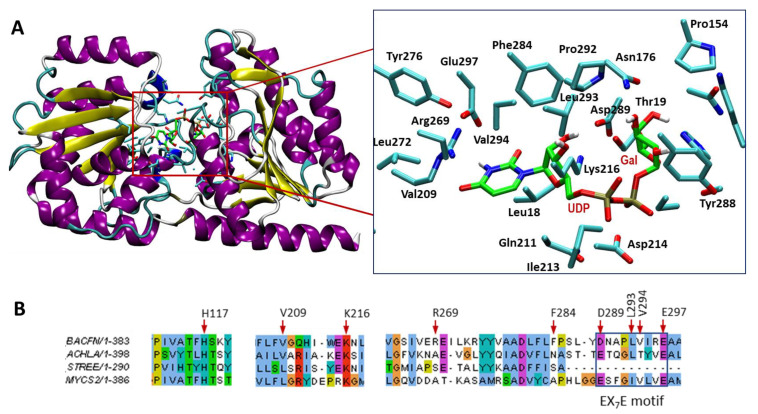
(**A**) Structural model of αCerGal_GT with bound UDP-Gal, generated by docking UDP-Gal into the AlphaFold model of the free enzyme (retrieved from Uniprot A0A380YRQ3). Right, magnification of the donor binding site, showing the amino acid side chains of first-shell residues interacting with UDP-Gal. (**B**) Sequence alignment of regions surrounding donor binding residues, GT4 glycolipid synthases from Bacteroides fragilis (αCerGal_GT, top), Acholeplasma laidlawii, Streptococcus pneumoniae and Mycolicibacterium smegmatis.

Based on the three-dimensional AlphaFold model of the free enzyme, a preliminary structural analysis was performed to evaluate the topology of the active site and identify residues interacting with the substrate. The αGalCer_GT amino acid sequence was aligned with GT4 enzymes with a solved crystal structure (see Supporting Information, Table S1 and Figure S4). Likewise, the AlphaFold model of αGalCer_GT was structurally aligned with the 3D structures of solved GT4 enzymes (Figure S5). αGalCer_GT does not differ from the typical GT-B fold of GT4 enzymes, comprising two separate β/α/β Rossmann-fold domains that form an inter-domain substrate-binding crevice. To locate the UDP-Gal donor binding site in the enzyme 3D model structure, docking experiments were performed, highlighting first-shell amino acid residues (Figure 11).

The closest protein with a solved X-ray structure is the phosphatidylinositol mannosyltransferase (PimA) from *Mycolicibacterium smegmatis* (Uniprot: A0QWG6) with 42.4% similarity and 24.4% identity (Figure S6). The structural alignment of the modeled αGalCer_GT with UDP-Gal ligand with the 3D structure of a complex PimA with bound GDP-Man substrate [34] allowed a comparison of the similar binding site topology (Figure S7) and first-shell amino acid residues in the binding site, although it should be taken as a tentative model due to the relatively low sequence identity between both proteins. Residues of αGalCer_GT interacting with the UDP nucleotide moiety comprise Val209, Arg269, Val294 and Glu297 (uracil), Lys216, Phe284 and Leu293 (ribose), whereas Thr19, His117, and Asp289 interact with the galactose unit (Figure 11). Acceptor docking experiments have not been attempted because the 3D enzyme model corresponds to the free enzyme, and a domain closure is expected upon acceptor binding, as is often observed in GT enzymes with a GT-B fold [35].

## 3. Discussion 

The *B. fragilis* genome contains 83 putative glycosyltransferases annotated in the CAZY database, from which GT sequences belonging to the GT4 family were selected, since it is the only retaining GT family with characterized glycolipid synthases using ceramide or diacylglycerol as acceptors. Only BF9343_3149, previously identified by Ito and coworkers [14], proved to have galactosylceramide transferase activity. 

Family 4 of glycosyltransferases is a broad family that comprises many different activities. The common features of the family are the retaining mechanism of action and the GT-B fold structure. αGalCer_GT has been reported to retain the α linkage of UDP-Gal in the αGalCer product, which matches with the mechanism of action of this family [14]. Likewise, sequence analysis shows two clear domains, which represent both domains of a GT-B structure. αGalCer_GT conserves the typical EX_7_E motif essential for activity in GT4 enzymes [36], but with an Asp for the first Glu (Figure 11B). 

The full-length protein was mainly expressed as an insoluble protein, but fused with SUMO it significantly improved the solubility. Purification by affinity chromatography (Step-tagged protein) afforded pure enzyme in about 0.6 mg per liter of culture. The purified protein, however, proved to be rather unstable, with a loss of activity in 24 h (Figure 6), and all enzymatic reactions were carried out within a time window of 2 h after enzyme purification. This instability is an important issue that should be addressed in further work to improve stability and enable applications in enzymatic synthesis. 

αGalCer_GT was shown to be a non-processive GT, since a single product was obtained with 100% conversion (Figure S2). It prefers UDP-Gal over UDP-Glc as a donor substrate (14.5-fold higher specific activity for UDP-Gal, Figure S3) and has no activity with UDP-GalNAc, UDP-GlcA, UDP-GlcNAc or GDP-Man, as reported [14]. It shows maximal activity at pH 7.3 and 30–35 °C and is fully inactive at 55 °C (Figure 8), indicating poor thermostability, in agreement with the observed instability upon storage even at 4 °C.

GT enzymes with a GT-A fold are metallodependent enzymes and exhibit the conserved DXD motif that binds a metal cation, which facilitates leaving group departure by coordinating the phosphate groups of the sugar nucleotide donor. In contrast, GT-B enzymes do not have the DXD signature but instead use positively charged side chains and/or hydroxyls and helix dipoles to facilitate leaving group departure during catalysis [37]. Metal cations are not essential for enzymatic activity, but in some cases reaction rates are accelerated by certain metal cations [38]. In αGalCer_GT, there is no evidence of metal cation requirement. EDTA did not reduce enzyme activity, the addition of Mg^2+^ or Mn^2+^ did not significantly affect the activity, but Zn^2+^ inactivated the enzyme (Figure 7). However, related GT4 glycolipid synthases such as GlcAT from *Z. mobilis* and α-monoglucosyldiacylglycerol synthase from *A. laidlawii* did show metallodependent activity, despite the lack of a DXD motif [14,39].

As for lipid acceptors, Okino et al. [14] showed that ceramide with a C12 chain length (C12-Cer-NBD) was the preferred acceptor, whereas 50% activity was observed with C6-Cer-NBD and no activity was detected with diacylglycerol (DG-NBD). However, the conditions that explain how the lipidic acceptor substrate was presented were not detailed. Other glycolipid synthases are membrane-associated proteins, i.e., monoglucosyldiacylglycerol synthases from *A. laidlawii* [39] and *Streptococcus pneumoniae* [25], processive MG517 from *Mycoplasma genitalium* [32] or PimA from *M. smegmatis* [34], which require anionic lipids for activation and being active with the lipid acceptor presented in mixed micelles. Our results indicate that αGalCer_GT is fully active with the ceramide acceptor solubilized with BSA, but activity was largely reduced when using ceramide in lipid vesicles, and no activation by anionic lipids was observed (Figure 10). Furthermore, when ceramide was solubilized with BSA but there were DOPC vesicles present in the reaction, enzyme activity was also substantially reduced. It seems that the enzyme is not membrane-associated, since the presence of detergents in the lysis buffer when extracting the protein did not improve protein solubilization. Altogether, these results suggest that the enzyme takes the lipidic acceptor presented by a lipid–protein complex rather than from membranes, but it requires further studies to elucidate the mechanism.

In conclusion, we report the biochemical characterization of the first recently discovered GT enzyme synthesizing α-galactosylceramide, a relevant non-mammalian glycolipid with great interest as an effector in immune responses and with applications as a vaccine adjuvant.

## 4. Materials and Methods 

### 4.1. In Silico Analysis

The CAZy database [23] was used to search for glycosyltransferase families in *Bacteroides fragilis*. Protein sequences were obtained from the UniProt database. Jalview was used for sequence alignments using MAFFT and TCoffee algorithms [40], with a neighbor-joining phylogenetic tree calculated using BLOSUM62 scores as a distance metric. A protein homology search was carried out using BLAST tools from NCBI and UNIPROT. 

### 4.2. Strains, Plasmids and Reagents

DH5α *E. coli* cells were used for cloning and molecular biology work. The cells were cultured in LB broth with the appropriate antibiotic at 37 °C, either on liquid culture or agar plates. BL21(DE3) Star *E. coli* cells were used for protein expression. pET28a and pET22b were from Novagen (Merck, Darmstadt, Germany). Ceramide-C6-NBD (Cer-NBD) (N-[6-[(7-nitro-2-1,3-benzoxadiazol-4-yl)amino]hexanoyl]-D-erythro-sphin-gosine) was provided by the synthesis facility at the Institut de Química Avançada de Catalunya, Barcelona, Spain. UDP-Galactose sodium salt was obtained from Merck. 

### 4.3. Cloning of B. fragilis GT Sequences into E. coli Expression Vector

(a) pET28a vector-Protein sequences were obtained from the UniProt database and then translated to DNA code optimized for *E. coli*. Synthetic sequences were obtained from GeneArt (ThermoFischer Scientific, Waltham, MA, USA) and cloned using restriction enzymes NcoI and XhoI into pET28a vector to obtain the expression vectors pET28a-HisTag-BF9343_XXXX. Positive transformants were selected on LB plates with 50 µg/mL of kanamycin. Successful plasmid constructions were verified by Sanger sequencing.
(b) pET22b-Strep-SUMO vector-Sequence BF9343_3149 was later cloned into a pET22b vector containing a N-terminal fused Strep-tag and SUMO protein. Cloning was carried out by Circular Polymerase Extension cloning (CPEC), amplifying the linear vector and the insert containing BF9343_3149 with primers sharing homologous regions, and CPEC reaction to obtain pET22b-SUMO-BF9343_3149-StrepTag. Positive transformants were selected on LB plates with 100 µg/mL of ampicillin. Successful plasmid construction was verified by Sanger sequencing.

### 4.4. Expression of GT Candidates and αGalCer_GT in E. coli

pET28a-HisTag-BF9343_XXXX.-BL21(DE3)Star cells harboring pET28a containing *B. fragilis* GT sequences were cultured in 300 mL of LB containing kanamycin at a final concentration of 50 µg/mL, inoculated at an initial OD of 0.05 and incubated at 37 °C until OD 1. At this point, protein expression was induced by adding IPTG to a final concentration of 1 mM, and cultures were incubated at 25 °C for 4 h. The cells were then harvested at 5000× *g* for 25 min and washed with 15 mL of NaCl 0.9%. The cell pellets were kept at −20 °C until use. 

pET22b-SUMO-BF9343_3149-StrepTag.-BL21(DE3)Star cells harboring pET22b-SUMO-BF9343_3149-StrepTag were grown in 600 mL of LB containing ampicillin at a final concentration of 100 µg/mL, inoculated at an initial OD of 0.05 and incubated at 37 °C until OD = 1. At this point, protein expression was induced by adding IPTG to a final concentration of 0.1 mM, and cultures were incubated at 30 °C for 4 h. The cell pellets were obtained as previously described.

### 4.5. Protein Purification by Strep-Tag Affinity Chromatography

Frozen cell pellets from the 600 mL cultures were thawed to room temperature and resuspended in 15 mL of lysis buffer (50 mM of Na_2_HPO_4_, 150 mM of NaCl, pH 7.5) supplemented with 1mM of PMSF (phenylmethylsulfonyl fluoride). The cells were disrupted by sonication in a Soniprep 150 sonifier at 4 °C (10 min, 10 s ON/20 s OFF, 50% amplitude). The lysate was centrifuged at 25,000× *g* for 60 min. The supernatant was recovered and filtered (0.45 μm) before purification with a StrepTrap HP 1 mL column (GE Healthcare, Chicago, IL, USA). The protein was eluted with lysis buffer with 2.5 mM of d-desthiobiotin. Eluted fractions were combined and dialyzed using Amicon Ultra-15 10 kDa (Millipore) with lysis buffer. The final retained fraction was recovered, and volume was adjusted to 1 mL with lysis buffer. Protein was quantified using the Bradford assay. 

### 4.6. Galactosylceramide Transferase Activity Assay 

Glycolipid synthase activity from fresh cell-free extracts or purified enzyme was measured with Cer-NBD as an acceptor and UDP-Gal (or UDP-Glc) as a donor by HPLC with fluorescence detector (HPLC 1200 Agilent with fluorescence detector, excitation wavelength at 458 nm and emission at 530 nm, Nova-pak C18 column, flowrate of 1 mL·min^−1^, eluent 75% acetonitrile in water), as described by Orive et al. 2020 [41]. Cer-NBD was solubilized with BSA at an equimolar concentration or using mixed vesicles with DOPC or/and DOPG, as explained below. Product (αGalCer-NBD) was quantified from the chromatograms from the relative areas of substrate and product:

[αGalCer-NBD]= [Cer-NBD]_0_ × Area_αGalCer-NBD_/(Area_Cer-NBD_ + Area_αGalCer-NBD_).

(a) Ceramide-NBD with BSA: Reactions were performed with 25 µM of Cer-NBD, 25 µM of BSA, 1.25 mM of UDP-Gal, phosphate buffer (50 mM of Na_2_HPO_4_, 150 mM of NaCl, pH 7.5) and the appropriate amount of enzyme in a 200 µL final reaction volume. It was preincubated at 37 °C for five minutes and the reaction was started by adding UDP-Gal. Sampling time was adjusted depending on enzyme concentration. Aliquots were withdrawn and mixed with methanol (MeOH:sample 8:2) and centrifuged to eliminate debris. The samples were then analyzed by HPLC. Chromatographic peaks were assigned by co-injection with independent standards. Initial rates were obtained from the linear progress curve of product formation. Initial rates were determined from triplicate assays.
(b) Ceramide-NBD in vesicles: Ceramide-NBD, DOPG and/or DOPG (matrix lipid) were dissolved in chloroform in a glass vial using the desired amounts for each experiment, as indicated. Chloroform was evaporated under a N_2_ stream and the lipid mixture dried under vacuum for 2 h. When the reactions were performed, the lipid film was solubilized to homogeneity in 80 µL of phosphate buffer solution (50 mM of Na_2_HPO_4_, 150 mM of NaCl, pH 7.5) by extensive vortexing and ultrasound bath for 5 min, and kept on ice for 1 min. This cycle was repeated 8 times. Vesicle formation was analyzed using ZetaSizer (Malvern Panalytical, Malvern, UK). For the enzymatic reaction, 25 µL of enzyme was added to 60 µL of freshly prepared vesicles solution. The mixture was kept for 30 min on ice and preincubated for 5 min at 37 °C. The reaction was started by adding 12.5 µL of UDP-Gal and incubated at 37 °C. The reaction conditions were: 1.25 mM of UDP-Gal, 25 µM of Cer-NBD, DOPC/DOPG vesicles (condition 1:1 mM of DOPC; 2:1 mM of total DOPC and DOPG (40 mol % DOPG); 3:1 mM of DOPG, phosphate buffer (50 mM of Na_2_HPO_4_, 150 mM of NaCl, pH 7.5), and enzyme (0.92–2.75 µM) in a 200 uL reaction volume. Aliquots were withdrawn every 30 min for 90 min, stopped as before and analyzed by HPLC.

### 4.7. Kinetic Parameters

UDP-Gal (0.1–2.5 mM) and ceramide-NBD (5–300 µM) with an equimolar amount of BSA were individually varied, maintaining constant the other components. Initial rates vs. donor or acceptor concentrations were fitted to a Michaelis–Menten equation with substrate inhibition by non-linear regression, using Prism 8 software (GraphPad, San Diego, CA, USA), from which the kinetic parameters were derived. 

### 4.8. Effect of Metal Cations

Tris buffer was used when analyzing metal binding to avoid precipitation of divalent cations. The cell pellet was resuspended with 20 mM of Tris buffer with 200 mM of NaCl, 1 mM of EDTA and 1 mM of DTT at pH 7.5 supplemented with 1 mM of PMSF. Enzyme purification was carried out following the same protocol as before, but with lysis buffer, 20 mM of Tris buffer and 200 mM of NaCl at pH 7.5. The reactions were performed with 25 µM of Cer-NBD, 25 µM of BSA, 1.25 mM of UDP-Gal, the appropriate amount of enzyme, Tris buffer (20 mM of Tris, 150 mM of NaCl, pH 7.5) with or without EDTA (200 mM), and the addition of metal ions (0.25–1 mM) in a 200 µL final reaction volume. It was preincubated at 37 °C for five minutes, and the reaction was started by adding UDP-Gal.

### 4.9. Modeled 3D Structure and Ligand Docking

The AlphaFold model of *B. fragilis* αGalCer_GT was obtained from Uniprot A0A380YRQ3. UDP-Gal structure was taken from PDB 5M7D. The complex αGalCer_GT·UDP-Gal was generated by docking with AUTODOCK VINA [42]. Both the protein and ligand structure were first parametrized with AutoDockTools4 [43]: polar hydrogens were added, Auto-Dock4.2 atom typing was used, and Gasteiger partial charges were computed. All the rotatable bonds of the ligands were considered free during the docking calculations, whereas the whole protein structure was kept fixed. The docking search space was confined in a box centered in the active site. The exhaustiveness level was set to 24, and 20 binding modes of the ligands were generated. Only low-energy binding poses were considered for analysis. Pictures of the complexes were generated with VMD [44].

## Data Availability

The data that support the findings of this study are available from the corresponding authors upon reasonable request.

## Supplementary Materials

The following are available online at https://www.mdpi.com/article/10.3390/ijms232213975/s1, Reference [45] is cited in the Supplementary Material.
