# Supplementary material for: Characterization of a Glycolipid Synthase Producing α-Galactosylceramide in Bacteroides fragilis
Source: Int J Mol Sci. 2022 Nov 12;23(22):13975. doi: 10.3390/ijms232213975 (PMC9692976; doi:10.3390/ijms232213975)

Supporting Information

Characterization of a glycolipid synthase producing α-galactosylceramide in *Bacteroides fragilis*

Marc Caballé, Magda Faijes* and Antoni Planas*

Laboratory of Biochemistry, Institut Químic de Sarrià, University Ramon Llull, Barcelona, Spain

Figure S1. Sequence alignment of annotated GT4 enzymes from *Bacteroides fragilis*.

Figure S2. Time-course reaction monitoring of galactosyltransferase activity of purified αGalCer_GT.

Figure S3. αCerGal_GT activity on UDP-Gal and UDP-Glc donor substrates.

Table S1. GT4 enzymes with solved crystal structure.

Figure S4. Sequence alignment of GT4 enzymes with solved X-ray structure.

Figure S5. Structural alignment of selected GT4 enzymes.

Figure S6. Sequence Similarity Matrix of GT4 sequences with solved X-ray structure.

Figure S7. Structural superposition of the modeled αGalCer_GT with bound UDP-Gal and PimA from *Mycolicibacterium smegmatis* with bound GDP-Man.

Figure S1. Multiple sequence alignment of annotated GT4 enzymes from *Bacteroides fragilis* and query sequences of monoglucosyldiacylglycerol synthases from *Streptococcus pneumoniae* [UniProt O06453] and *Acholeplasma laidlawii* [Uniprot Q93P60] (first and last sequences in the alignment, respectively). Alignment with T-Coffee using JalView (40).

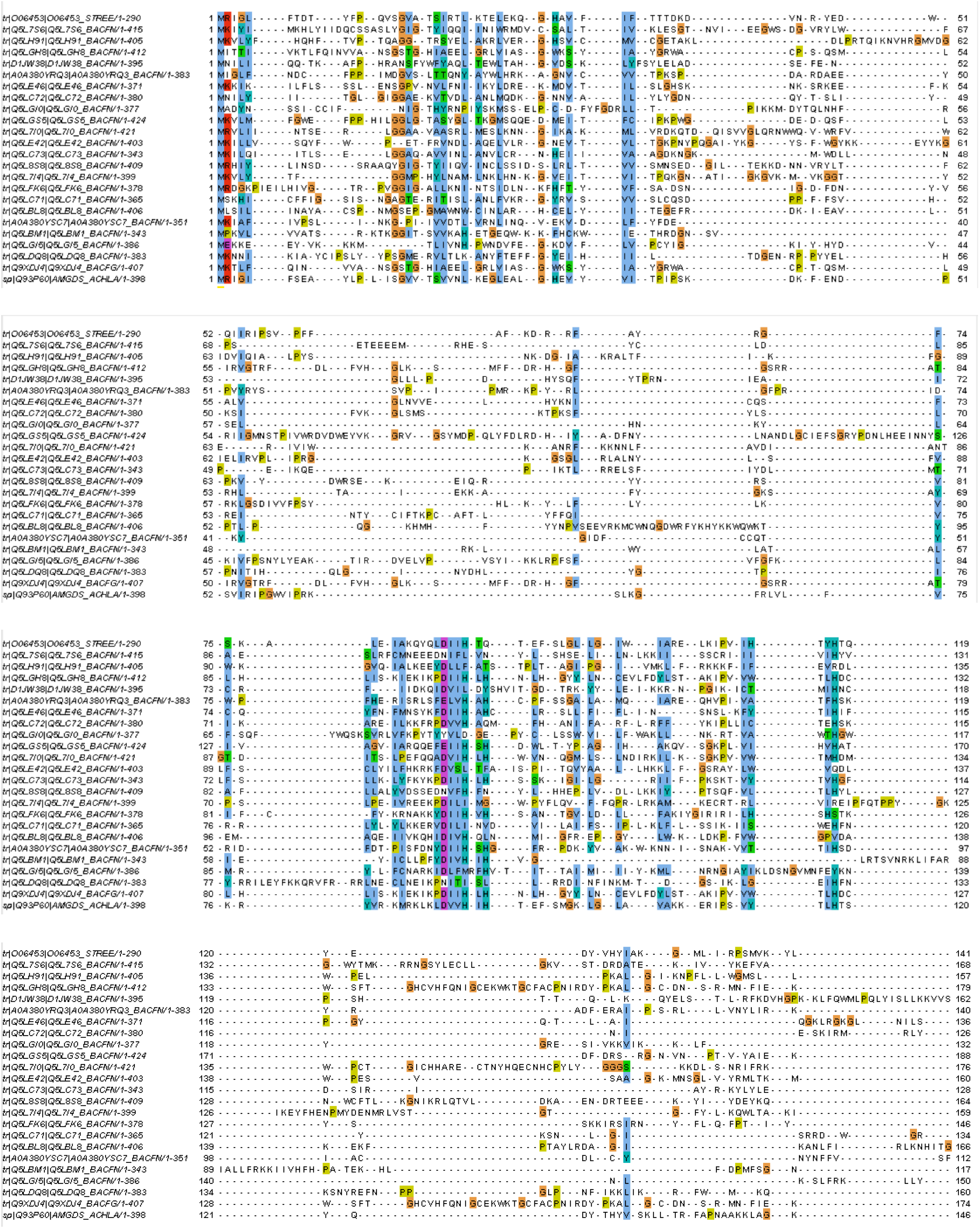

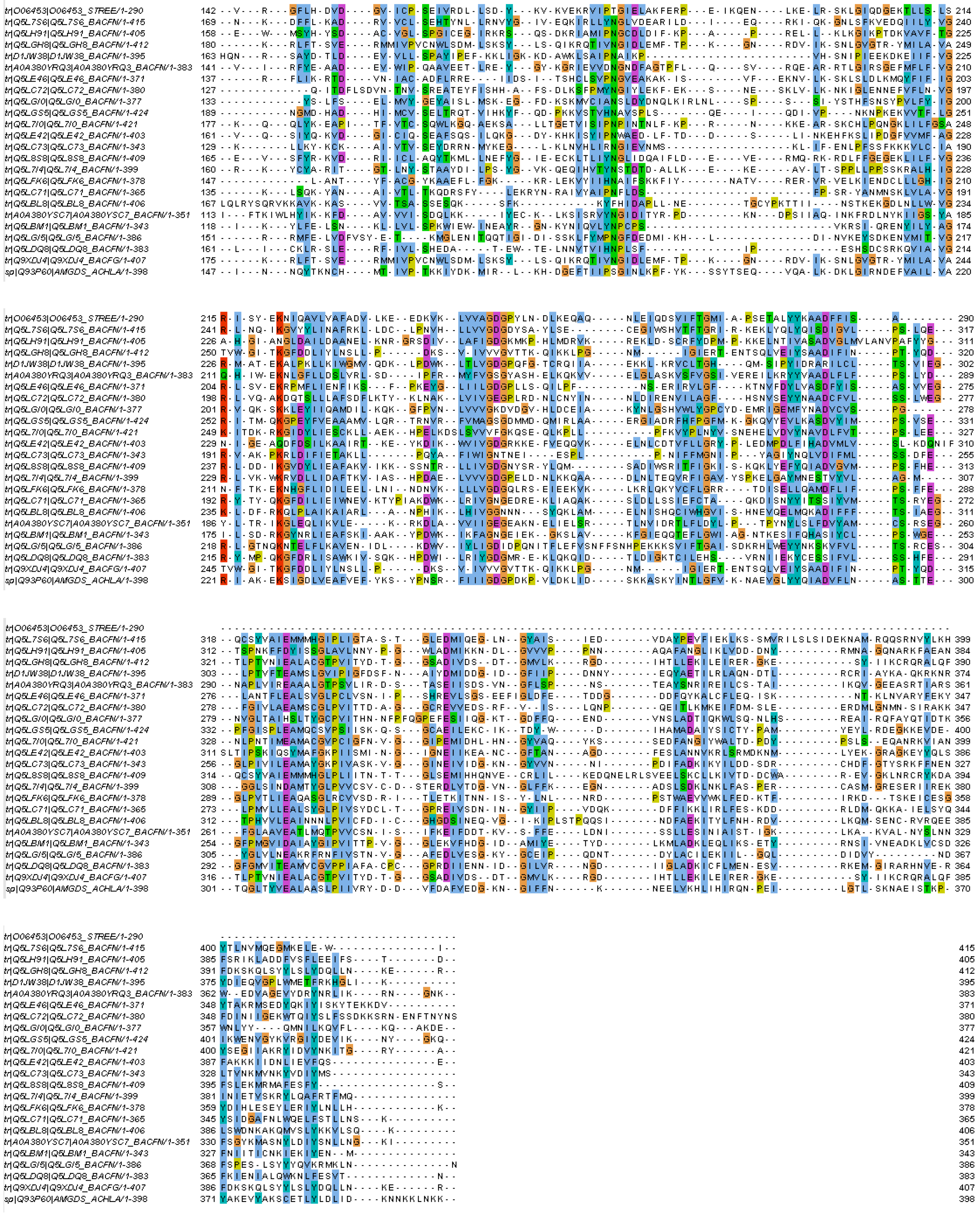

Figure S2. Time-course reaction monitoring of galactosyltransferase activity of purified αGalCer_GT. The reaction was carried out with 25 µM of Cer-NBD in the presence of equimolar BSA, 1.25 mM of UDP-Gal and 2.75 µM enzyme in phosphate buffer at pH 7.5 and 37 ºC.

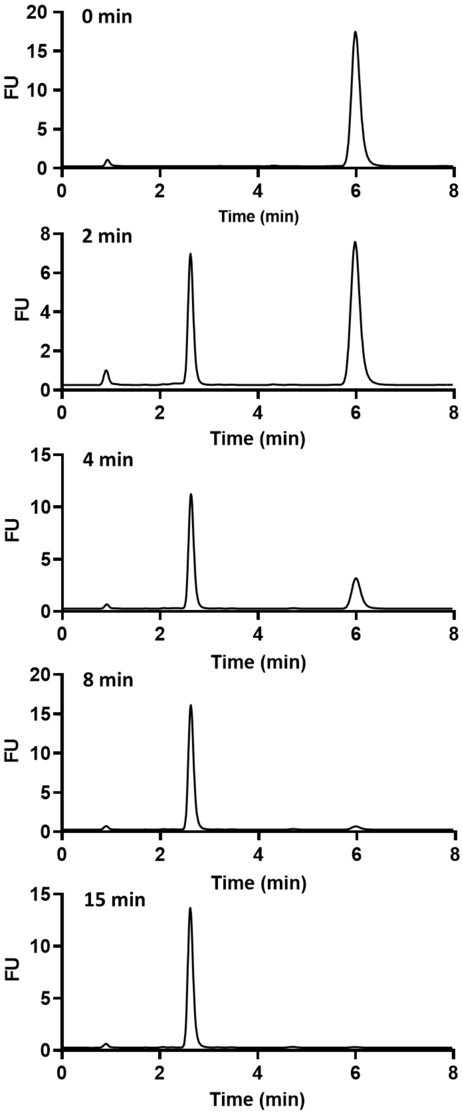

Figure S3. αCerGal_GT activity on UDP-Gal and UDP-Glc donor substrates. Reactions conditions: 1.25 mM UDP-Gal or UDP-Glc, 25 µM Cer-NBD in the presence of equimolar BSA, different enzyme concentrations (520 nM to 1.04 µM) at pH 7.5 and 37 ºC. Specific activities: UDP-Gal, 0.031±0.003 s^{-1}, UDPGlc, 0.0021±0.0003 s^{-1}.

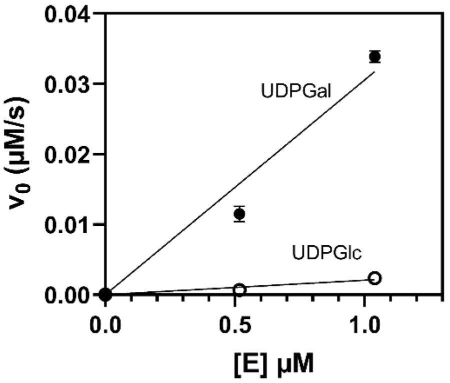

Table S1. GT4 enzymes with solved crystal structure (October 2022). Proteins included in the sequence alignment (Figure S3) and structural alignment (Figure S4) are indicated in columns "Sequence alignment" and "Structural alignment", respectively.

Name	Organism	Uniprot	PDB	Ligand	Resolution	Sequence alignment[1]	Structural alignment[2]
**UDP-GlcNAc L-malate α-N-acetylglucosaminyltransferase**	Bacillus anthracis str. Ames	Q81ST7	2JJM	-	3.10	YES	YES
**UDP-GlcNAc L-malate α-N-acetylglucosaminyltransferase**	Bacillus anthracis str. Sterne	Q81ST7	3BMO	UDP and L-malate	3.31	-	REMOVED
**UDP-GlcNAc L-malate α-N-acetylglucosaminyltransferase**	Bacillus subtilis subsp. subtilis	P42982	5D00	N-acetylglucosaminyl-malate and UMP	2.15	YES	YES
**UDP-GalNAc: α-1,4-N-acetylgalactosyltransferase**	Campylobacter jejuni subsp. jejuni	O52905	6EJI	-	2.30	YES	YES
**UDP-GlcNAc: 1L-myo-inositol-1-P α-N-acetylglucoaminyl-transferase**	Corynebacterium glutamicum ATCC 13032	Q8NTA6	3C48	-	2.10	YES	YES
			3C4V	UDP and 1L-INS-1-P.	2.60	-	YES
**α-mannosyltransferase**	Corynebacterium glutamicum	Q8NNK8	3OKA	-	2.20	YES	YES
**UDP-GalNAc: GalNAc-PP-Und α-1,3-N-acetylgalactosaminyl-transferase (WbnH)**	Escherichia coli O86:H2	P0DMP6	4XYW	-	2.20	YES	YES
**UDP-Glc: L-glycero-D-manno-heptose II α-1,3-glucosyl-transferase I (WaaG,RfaG)**	Escherichia coli str. K-12 substr. MG1655	P25740	2IV7	-	1.6	YES	YES
**FTT1235c (LpcC)**	Francisella tularensis subsp. tularensis SCHU S4	Q5NFJ9	5I45	-	1.35	-	REMOVED
**sucrose phosphate synthase (SpsA)**	Halothermothrix orenii	B2CCB8	2R60	-	1.80	YES	YES
			2R68	P-(0-6)-β-D-Fruf-(2-1)-α-D-Glcp	2.40	-	YES
**cholesterol α-glucosyltransferase (HP0421)**	Helicobacter pylori 26695	O25175	3QHP	-	1.50	YES	REMOVED
**phosphatidylinositol mannosyltransferase**	Mycolicibacterium smegmatis MC2 155	A0QWG6	2GEJ	GDP-Man	2.60	YES	YES
			4N9W	-	1.94	-	YES
**ADP-Glc-dependent α-maltose-1-phosphate synthase**	Mycolicibacterium smegmatis MC2 155	A0R2E2	6TVP	-	1.90	YES	YES
**sucrose synthase**	Nitrosomonas europaea ATCC 19718	Q820M5	4RBN	-	3.05	REMOVED	REMOVED
**UDP-Glc: [heterocyst envelope polysaccharide] mannoside glucosyltransferase**	Nostoc sp. PCC 7120 = FACHB-418	Q8YQW3	4XSO	-	2.01	YES	YES
			4XSP	-	2.15	-	YES
			4XSR	UDP-Glc	2.39	-	YES
			4XSU	α-D-Glcp + UDP	2.48	-	YES
**RAF_ORF0434**	Rickettsia africae ESF-5	C3PN56	7MI0	-	2.90	NO	YES
**LPS 1,6-galactosyltransferase (RfaB)**	Salmonella enterica subsp. enterica serovar Typhimurium str. LT2	Q06994	5N80	UDP	1.92	YES	YES
			6Y6I	UDP	1.92	-	YES
**UDP-GlcNAc: teichoic acid α-N-acetylglucosaminyltransferase**	Staphylococcus aureus subsp. aureus 21178	A0A0H2WWV6	4X6L	UDP	3.19	REMOVED	REMOVED
			4X7M]	UDP-GlcNAc	2.40	-	-
			4X7R	α-GlcNAc-glycerol and UDP	2.15	-	-
**UDP-GlcNAc: teichoic acid α-N-acetylglucosaminyltransferase**	Staphylococcus aureus subsp. Aureus	A0A0D6HUA0	4WAC	-	2.40	REMOVED	REMOVED
		-	4WAD	UDP-GlcNAc	2.80	-	REMOVED

**Serine-O-α-N-acetylglucosaminyltransferase**	Staphylococcus aureus subsp. aureus	A0A0H2URG7	7EC1	-	1.85	REMOVED	REMOVED
			7EC3	β-D-GlcpNAc	2.50	-	-
			7EC6	ASP-SER-ASP	1.90	-	-
			7VFL	β-D-GlcpNAc	2.45	-	-
			7VFM	UDP and SD peptide-binding form	2.28	-	-
**UDP-GlcNAc: L-malate α-N-acetylglucosaminyltransferase**	Staphylococcus aureus subsp. Aureus	A0A068A5A2	6D9T	UDP	2.00	YES	YES
			6N1X	α-D-GlcpNAc	2.35	-	YES
**UDP-GlcNAc: [Ser/Thr] O-N-acetylglucosaminyltransferase**	Streptococcus gordonii M99	Q9AET5	5E9T	-	2.92	REMOVED	REMOVED
			5E9U	β-D-GlcpNAc	3.84	-	-
**UDP-GlcNAc: protein [Serine] α-N-acetylglucosaminyltransferase**	Streptococcus pneumoniae TIGR4	A0A0H2URG7	4PQG	β-D-GlcpNAc	2.00	REMOVED	REMOVED
**eurekanate-attachment enzyme (AviGT4)**	Streptomyces viridochromogenes TUE57	Q93KV2	2IUY	-	2.1	YES	YES
**UDP-Glc: tetrahydrobiopterin α-glucosyltransferase**	Synechococcus elongatus PCC 7942 = FACHB-805	Q31LX1	5ZE7	-	1.99	YES	YES
			5ZFK	UDP-BH2 complex	1.75	-	YES
**sucrose-phosphate synthase (TeSPS; Tll1590)**	Thermosynechococcus vestitus BP-1	Q8DIJ5	6LDQ	-	1.92	REMOVED	REMOVED
**UDP-Glc: α-1,2-glucosyltransferase (Tll1591)**	Thermosynechococcus vestitus BP-1	Q8DIJ4	7FG9	-	2.66	YES	YES

[1] Sequence alignment. A total of 26 GT4 were first selected. 6 were removed because of poor alignment. YES are the 18 sequences included in the sequence alignment in Figure S3.
[2] Structural alignment. Protein sequences removed from the sequence alignment were also removed from the structural alignment. Additionally, structures labeled “REMOVED” in red were also discarded because of missing residues in the X-ray structure. Structural alignment in Figure S5.

Figure S4. Sequence alignment of GT4 enzymes. Selected sequences from Table S1 (18 sequences + BF9343_3149 (Uniprot A0A380YRQ3, *Bf*αGalCer_GT, on top). Alignment with T-Coffee using JalView (40).

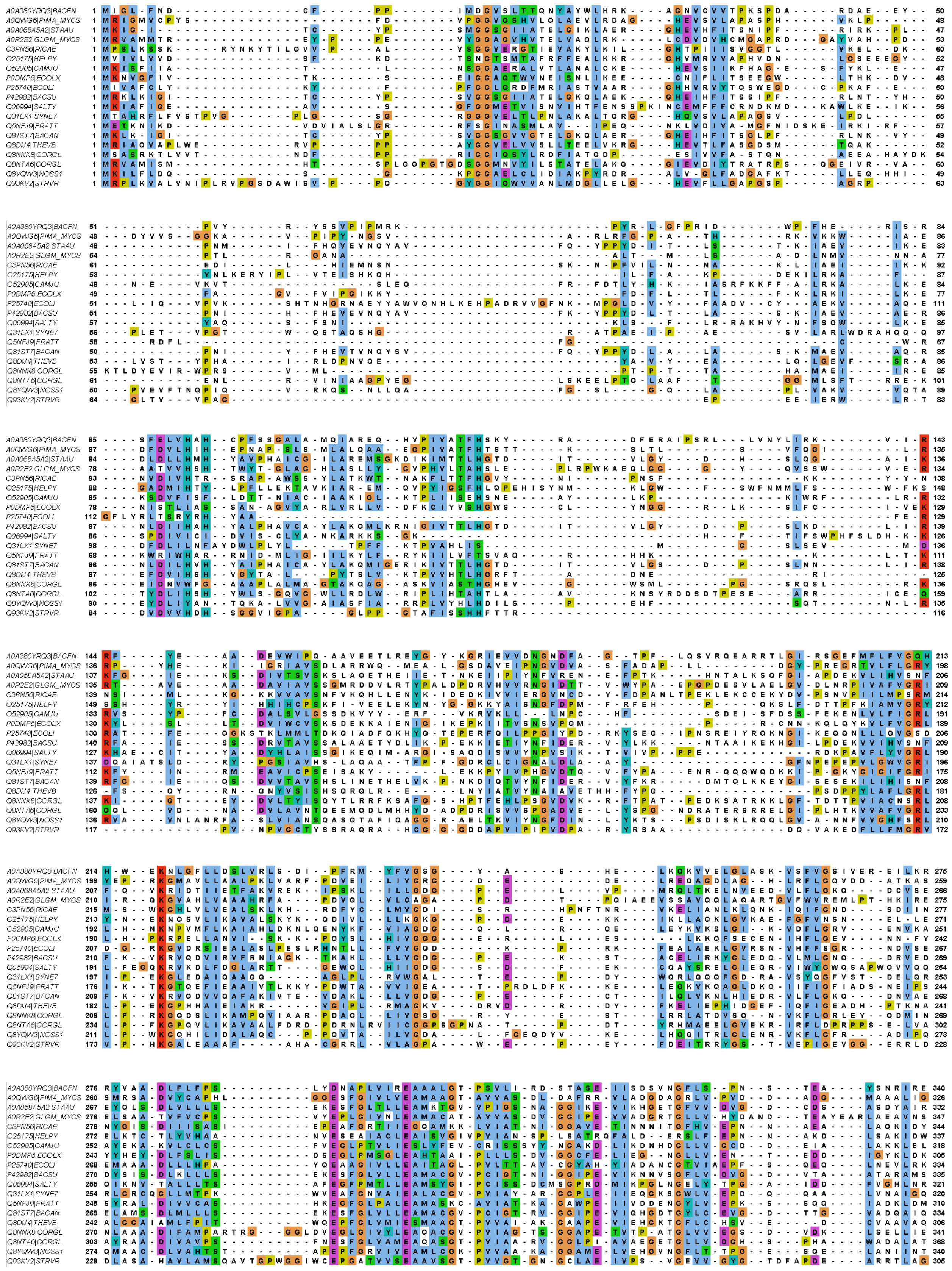

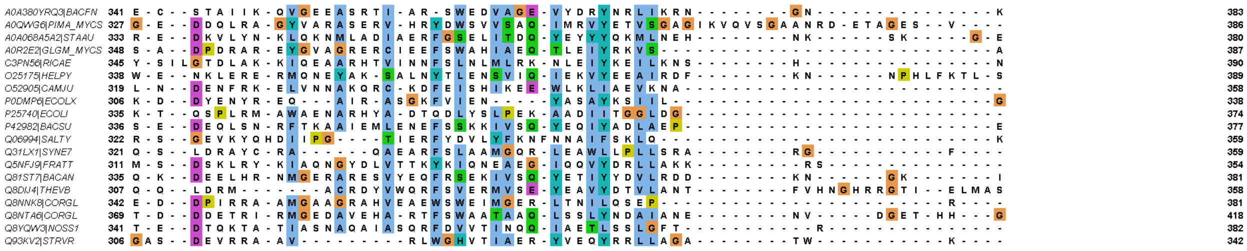

Figure S5. Structural alignment of 10 selected GT4 enzymes (2JIM, 5D00,3OKA, 2IV7, 2GEJ, 4XSU, 5N80, 6D9T, 2IUY, 5ZFK from Table S1) and the αGalCer_GT (orange). Figures generated with VMD (44).

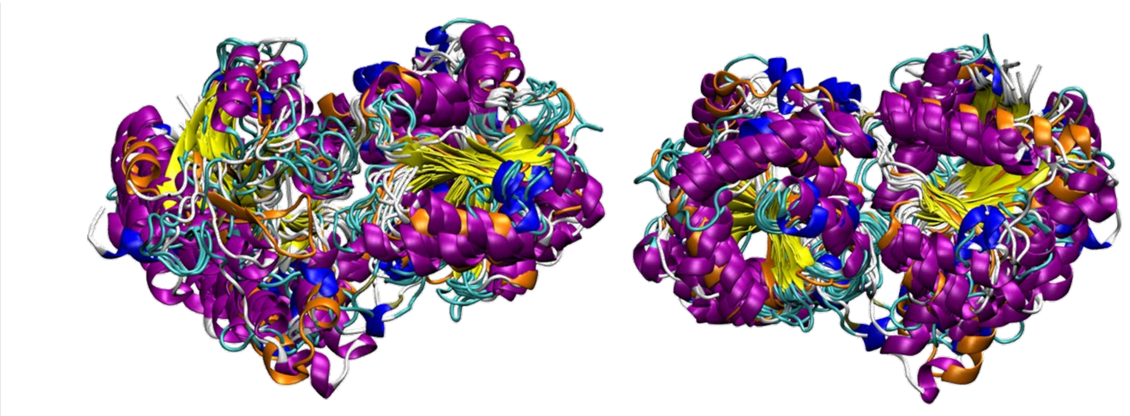

Figure S6. Sequence Similarity Matrix of GT4 sequences with solved X-ray structure and aligned in Figure S4. *Bf*αGalCerGT (Uniprot A0A380YRQ3) as sequence 1. Matrix generation with MatGAT (45). Below diagonal: similarity; above diagonal: identity, using BLOSUM62.

UNIPROT	1	2	3	4	5	6	7	8	9	10	11	12	13	14	15	16	17	18
1. A0A380YRQ3|BACFN		24,4	18,2	22,5	17,5	22,3	18,9	18,1	19,1	19,2	17,1	18,5	17,1	19,1	19,3	16,8	21,2	22,9
2. A0QWG6|PIMA_MYCS2	42,7		19,9	25,8	17,7	20	20,6	19,7	18,2	20,5	16	23,1	20,5	22,4	25,8	23,5	23,5	21
3. A0A068A5A2|STAAU	39,2	42,5		17	22,2	20,2	19,7	21,6	18,6	54,6	21,7	20,6	18,7	55,2	21,9	20,3	21,1	21,8
4. A0R2E2|GLGM_MYCS2	40,1	41,6	38,8		20,2	17,3	19,5	19,1	20	23,8	15,9	21,4	19,1	23,5	22,6	23,5	26,9	21,4
5. C3PN56|RICAE	39,7	39,2	43,6	40		17,6	19,8	18,7	16,9	25,1	14,5	19,6	20,7	24,4	20,7	22	20,1	20
6. O25175|HELPY	41,9	37,5	38,8	39,3	39		19,8	17,7	18,5	18,3	16,9	21,1	20,8	19,3	20,4	18,4	19,1	18,1
7. O52905|CAMJU	36	36,8	39,2	37,2	40	37		22,6	17	21	18,8	20,7	16,6	20,8	18,2	18,8	18,1	16,2
8. P0DMP6|ECOLX	34,5	38,3	41,1	35,1	35,4	35	41,9		17,2	20,2	21,4	18	19,5	20	18,9	22,1	19,4	21,1
9. P25740|ECOLI	38,6	36	35,8	38,8	40	37,8	38,5	32,6		18,1	17,3	20,4	16,7	19,4	18,6	21,7	21,1	20,8
10. P42982|BACSU	40,7	38,3	72,9	41,6	42,8	39,1	40,3	39	35,5		19	23	18,6	63,9	22,1	22,1	21,6	21,4
11. Q06994|SALTY	36,6	35,2	40,8	35,7	36,2	34,4	41,2	39,8	34,2	35		17,2	16,6	20,3	19,9	14,8	18,8	20,5
12. Q31LX1|SYNE7	34,2	38,1	36,6	34,9	36,7	37	39,6	35,4	36,4	37,9	35,4		19,6	20,2	28,3	22,8	20,4	24,4
13. Q5NFJ9|FRATT	37,6	38,3	38,2	38,8	37,9	40,4	41,3	36,7	39	39	39,6	38,4		19,7	18,9	20,7	21,7	20,9
14. Q81ST7|BACAN	42	42,2	75,1	42,9	43,8	37,3	38,6	37,5	37,8	79,3	36	35,2	40,4		25,3	23,5	23,6	21,2
15. Q8DIJ4|THEVB	34,5	39,9	39,7	38,5	37,7	39,6	40,2	37,7	35,6	41,4	40,1	47,1	37,2	44,6		17,3	22,5	21,2
16. Q8NNK8|CORGL	36,8	40,7	41,5	41,1	39,7	34,4	36,2	40,2	38,6	40,2	31,5	36	36,5	44,1	35,7		22,3	22
17. Q8NTA6|CORGL	39,7	40,4	40	42,6	39	35,9	37,1	34,7	39,5	36,4	35,2	36,4	40	43,3	38	38		20,8
18. Q8YQW3|NOSS1	38,6	38,6	40,6	39,3	41,8	36,2	36,1	39,5	37,7	40,1	38	40,6	41,4	42,9	39,5	42,1	38,5	
19. Q93KV2|STRVR	29,8	38,6	36,8	36,2	31,3	34,2	33,8	34,8	30,5	36,3	32	35,7	35	32	40,5	35,7	32,5	32,5

Figure S7. Structural superposition of the modeled αGalCer_GT with bound UDP-Gal (coloured by secondary structure) and PimA from *Mycolicibacterium smegmatis* with bound GDP-Man (PDB 2GEJ) (silver; GDP-Man in blue). Figure generated with VMD (44).

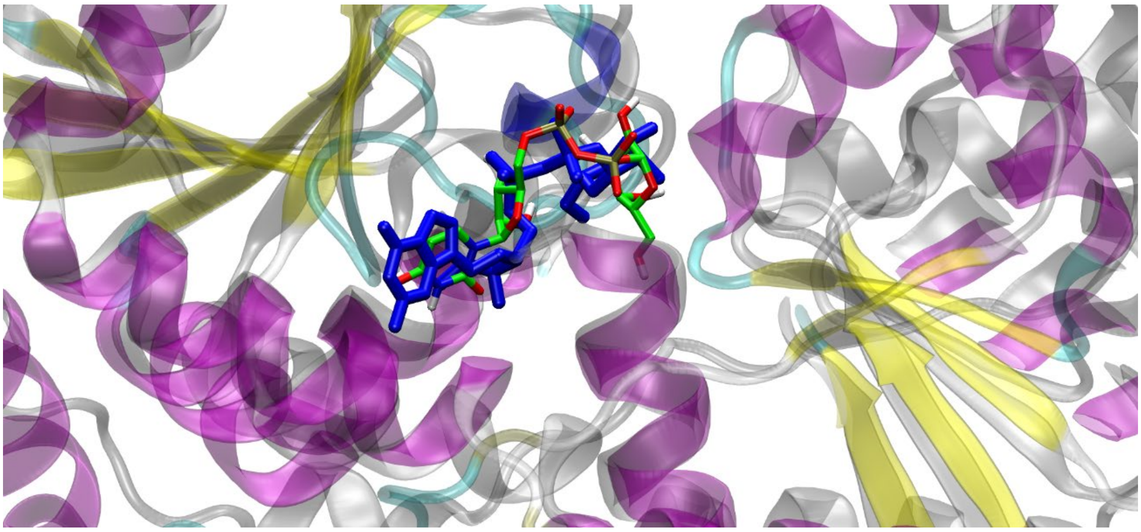

Supplement: Supplementary file 1 [file ijms-23-13975-s001.zip › ijms-2006748-supplementary.pdf]
